# Multicenter 30-Year Data on the 4-Mg Intravenous Dexamethasone Suppression Test in the Diagnosis of Cushing Syndrome

**DOI:** 10.1210/jendso/bvaf188

**Published:** 2025-11-21

**Authors:** Caroline Jung, Maresa M Derbyshire, Niyati Jauhar, Daniel Bennett, Duncan J Topliss, Reetu Gogna, Nikola Markovic, John R Burgess, Warrick J Inder

**Affiliations:** Department of Endocrinology and Diabetes, St Vincent's Hospital Melbourne, Fitzroy, VIC 3065, Australia; Department of Medicine, The University of Melbourne, Melbourne, VIC 3010, Australia; Department of Endocrinology and Diabetes, St Vincent's Hospital Melbourne, Fitzroy, VIC 3065, Australia; Department of Endocrinology and Diabetes, Alfred Health, Melbourne, VIC 3004, Australia; Department of Endocrinology and Diabetes, Alfred Health, Melbourne, VIC 3004, Australia; Department of Endocrinology and Diabetes, Alfred Health, Melbourne, VIC 3004, Australia; Department of Medicine, Monash University, Melbourne, VIC 3800, Australia; Department of Endocrinology and Diabetes, Royal Hobart Hospital, Hobart, TAS 7001, Australia; Department of Endocrinology and Diabetes, Royal Hobart Hospital, Hobart, TAS 7001, Australia; Department of Endocrinology and Diabetes, Royal Hobart Hospital, Hobart, TAS 7001, Australia; Department of Endocrinology and Diabetes, University of Tasmania, Hobart, TAS 7001, Australia; Department of Endocrinology and Diabetes, Princess Alexandra Hospital, Brisbane, QLD 4102, Australia; Department of Medicine, The University of Queensland, Brisbane, QLD 4072, Australia

**Keywords:** cortisol, intravenous dexamethasone suppression test, Cushing syndrome

## Abstract

**Context:**

The diagnosis and differential diagnosis of Cushing syndrome (CS) is often challenging.

**Objective:**

To evaluate the 4-mg intravenous dexamethasone suppression test (IVDST) to differentiate CS from normal subjects and subjects with low probability of CS (LPC), and define the cortisol responses in pituitary, adrenal, and ectopic adrenocorticotropin (ACTH) CS.

**Methods:**

Data from 140 patients with surgically confirmed Cushing disease (CD), 5 with ectopic ACTH syndrome (EAS), 26 with adrenal CS (AC), and 97 with LPC, from 4 tertiary hospitals between 1995 and 2024 were retrospectively evaluated. Thirty-two controls (normal and overweight/obese participants with or without type 2 diabetes) were previously studied. Dexamethasone was infused at 1 mg/h for 4 hours. Plasma cortisol and ACTH were measured at −60 minutes, −5 minutes, +3 hours, +4 hours, +5 hours and on Day 2 (+23 hours and +23.5 hours). Main outcome measures were the sensitivity and specificity of the IVDST for the diagnosis of CS.

**Results:**

Controls showed marked cortisol suppression across Days 1 and 2. In 17 of 97 patients with LPC, Day 2 cortisol overlapped with CS. Day 2 cortisol level of >130 nmol/L diagnosed CS with 97% sensitivity and 87% specificity.

**Conclusion:**

The IVDST is a highly sensitive second-line diagnostic test for CS. False negative results occurred when IVDST is performed during an eucortisolemic phase of cyclic CD. The specificity of 87% emphasizes the importance of long-term follow-up of LPC. The small number of EAS cases is a major limitation in the use of IVDST to differentiate ACTH-dependent CS.

Cushing syndrome (CS) is caused by chronic excessive exposure to glucocorticoids [1]. Endogenous CS is a rare disease, with an estimated incidence of 2 to 3 cases per million population [2]. The clinical manifestations of CS are highly variable, and the diagnosis can be challenging in mild, subclinical, or cyclic cases [3-7]. Furthermore, the differentiation between CS and pseudo-Cushing state (pseudo-CS) can be extremely difficult, given the overlap of clinical and/or biochemical features of hypercortisolism. Conditions that are common in the general population, such as obesity, depression, alcoholism, and diabetes mellitus, may cause pseudo-CS [1, 8], which was renamed as physiologic (non-neoplastic) hypercortisolism during the past decade [9]. The majority of the physiological states of hypercortisolism are mediated by hyperactivation of the hypothalamic-pituitary-adrenal (HPA) axis [9].

Numerous biochemical tests have been evaluated to establish the diagnosis of CS and to differentiate the etiology which can be categorized into adrenocorticotropin (ACTH)-dependent CS caused by pituitary (Cushing disease [CD]), or ectopic tumors (ectopic ACTH syndrome [EAS]), or ACTH-independent (adrenal Cushing's [AC]). Limitations of oral dexamethasone suppression testing (DST) include poor compliance and variable absorption of dexamethasone. To overcome these problems, various forms of the intravenous DST have been evaluated during the past 60 years, including 8-mg bolus [10], 5 µg/kg/h for 5 hours [11] and fixed dose infusions (1-1.5 mg/h) administered over 3 to 7 hours [12-20]. All the studies assessed the plasma cortisol response on the day of the infusion. Six studies [10, 11, 14, 18-20] have also assessed the cortisol response on the following morning (Day 2), including our previous study [19] on the 4-mg (1 mg/h over 4 hours) intravenous dexamethasone suppression test (IVDST).

This current study is an extension of our previous work [19], in which we have retrospectively evaluated the performance of the IVDST in 171 patients with clinically overt CS (140 patients with CD, 5 with EAS, and 26 with AC), compared to 97 with low probability of Cushing syndrome (LPC), from 4 tertiary hospitals between 1995 to 2024. The control group was drawn from our previous study [19]. The purpose of this study was to re-evaluate the Day 2 cortisol diagnostic criteria, the utility of ACTH profiles and to compare the responses to the IVDST in CS of various etiologies.

## Material and Methods

### Control Subjects

The 32 control subjects were recruited from the general community between 2007 and 2008. The inclusion and exclusion criteria and clinical characteristics of 3 subgroups of control subjects (normal weight [body mass index (BMI) < 25 kg/m^2^] or overweight or obese subjects [BMI >25 kg/m^2^] with or without type 2 diabetes mellitus), and the statistical basis for combining the results of these subgroups as the control group were previously published [19]. The mean age of the control group is included in Table 1.

**Table 1.** Thirty-two control subjects who were recruited for the prospective IVDST study between 2007 and 2008 [19], and a retrospective review of 523 patients who underwent IVDST between 1995 and 2024: inclusion criteria for the diagnosis of CD, EAS, AC, and LPC, exclusion criteria, and clinical characteristics

	Criteria for diagnosis or exclusion	Number of patients (M:F)	Age, years, mean ± SEM (range)
Control	Normal (BMI < 25 kg/m^2^) or overweight or obese subjects (BMI > 25 kg/m^2^) with or without Type 2 diabetes mellitus	32 (17:15)	52 ± 2 (31-70)
CD*^a^*	Patients who underwent IVDST before first pituitary operation and demonstrated surgically proven CD, defined as histological confirmation of a pituitary adenoma immunostaining for ACTH *and/or* remission of hypercortisolism postoperatively	140 (39:101)	42 ± 1 (14-85)
EAS	Clinical features of CS *and* neuroendocrine tumor outside the pituitary gland*^b^*	5 (1:4)	58 ± 6 (44-79)
AC	Low baseline ACTH levels *and* histological confirmation of adrenal tumor(s)	26 (4:22)	46 ± 3 (17-72)
LPC	Suspicion of possible CS*^c^* but subsequent follow-up over a 2-year period revealed no progression in the clinical features of CS.	97 (29:68)	47 ± 2 (17-90)
255 patients excluded from analysis	Insufficient information in the hospital medical records regarding the final diagnosis, either due to follow-up elsewhere, or less than 2 years follow-up at the hospital	159 (45:114)	50 ± 1 (16-84)
	Suspected CS based on the results of IVDST but the diagnosis not surgically proven*^d^*	34 (9:25)	
	Interfering medications (exogenous glucocorticoid, oral estrogen, anticonvulsant medications)	8 (2:6)	
	Patients who underwent IVDST after first pituitary operation	32 (7:25)	
	Mild autonomous cortisol secretion (MACS), defined as presence of adrenal lesion and abnormal 1-mg DST and suppressed basal ACTH level <2.2 pmol/L in patients with no overt features of CS	6 (1:5)	
	Adrenal incidentalomas, other than MACS	15 (4:11)	
	Subclinical CD, defined as a lack of clinical features of overt CS, but had abnormal 1-mg DST (plasma cortisol >50 nmol/L) before the pituitary operation and positive tumor ACTH immunoreactivity	1 (0:1)	

Abbreviations: AC, adrenal Cushing syndrome; ACTH, adrenocorticotropin; BMI, body mass index; CD, Cushing disease; CS, Cushing syndrome; EAS, ectopic adrenocorticotropin syndrome; F, female; IVDST, intravenous dexamethasone suppression test; LPC, low probability of Cushing syndrome; M, male; SEM, standard error of mean.

^a^In the CD group, 3 of 140 patients (2%) demonstrated cyclicity, defined as at least 2 peaks of hypercortisolism, interspersed with at least 1 trough of eucortisolism, based on screening tests for CS (24-hour urinary free cortisol and/or late-night salivary cortisol). In the CD group, 100 patients (71%) had microadenoma (defined as pituitary adenoma <10 mm demonstrated on MRI), 22 patients (16%) had macroadenoma (defined as pituitary adenoma 10 mm or greater demonstrated on MRI). MRI report was unavailable in 18 patients (13% of CD data).

^b^EAS tumor types: ovarian neuroendocrine small cell carcinoma, gastrinoma, neuroendocrine small cell lung carcinoma, bronchial carcinoid, ectopic corticotropin-releasing hormone (CRH) tumor.

^c^Suspicious clinical features in the LPC group included obesity, diabetes mellitus, hypertension dyslipidemia, depression, hirsutism, incidental adrenal or pituitary lesions

^d^22 patients did not undergo pituitary surgery, 1 patient did not undergo adrenal surgery, 8 patients underwent pituitary or adrenal surgery but did not fulfill the criteria for the diagnosis of CS and 3 patients underwent bilateral adrenalectomy.

### Cushing Syndrome and Low Probability of Cushing Syndrome

Cases of CS and LPC were drawn from a retrospective review of 523 consecutive patients who underwent IVDST between 1995 and 2024 at 4 tertiary hospitals in Australia: St. Vincent's Hospital, Melbourne (SVHM), Alfred Hospital (AH), Royal Hobart Hospital (RHH), and Princess Alexandra Hospital (PAH). Patients were referred for IVDST as a second-line diagnostic test after they had abnormal or discordant results in the first-line screening tests, such as 24-hour urinary free cortisol (UFC), 1-mg overnight DST, or late-night salivary cortisol (LNSC), performed by their referring doctor. Although incomplete data on screening test results prevented a comprehensive analysis, at least 12% of patients with CS were documented to have discordant results.

Final diagnoses were based on a review of hospital medical records, including histopathology results where available. The criteria for the diagnosis of CD (n = 140), EAS (n = 5), AC (n = 26), and LPC (n = 97) and clinical characteristics are shown in Table 1. The key criteria for classification of LPC (in whom the diagnosis of CS was not excluded based on initial screening tests) were absence of progression in the clinical features of CS, in particular, lack of development of specific signs, such as thin skin, spontaneous bruising, wide striae, or proximal myopathy, during a follow-up period of 2 years or more (range, 2-13 years). The exclusion criteria are shown in Table 1.

### Intravenous Dexamethasone Suppression Test

**Figure 1.**
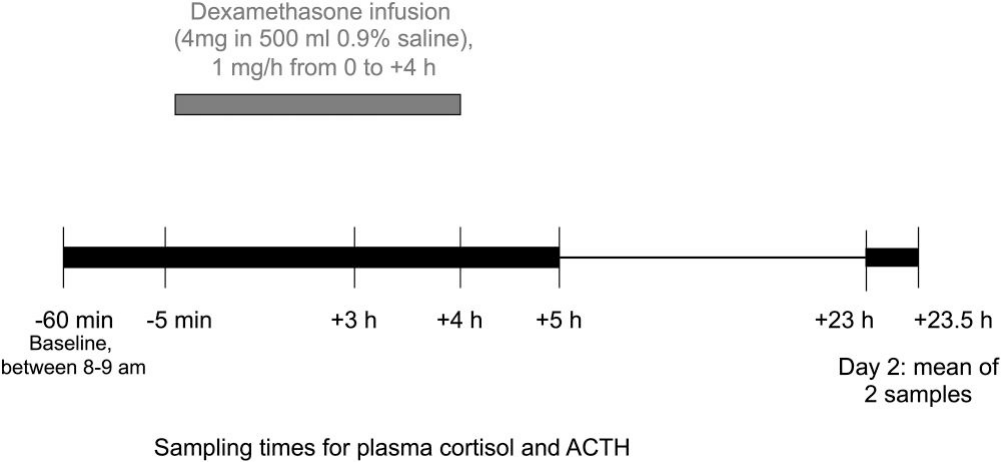
Outpatient IVDST protocol used between 1995 and 2024. Dexamethasone (4 mg) was diluted in 500 mL 0.9% saline and administered by an intravenous infusion at 1 mg/h from 0 to +4 hours. Plasma cortisol and ACTH were measured on the day of the dexamethasone infusion (Day 1) at −60 minutes (baseline sample drawn between 8 to 9 Am), −5 minutes, +3 hours, +4 hours and +5 hours. Two samples were taken on the following morning (Day 2) 30 minutes apart at +23 hours and +23.5 hours, and the mean values were used for the Day 2 cortisol and ACTH analysis.

The IVDST was performed in control subjects in the outpatient endocrine procedures unit at St Vincent's Hospital, Melbourne, according to a protocol previously published [19], which is shown in Fig 1. The IVDST was performed in patients with CS and LPC in the outpatient endocrine procedures units by endocrine nurses at the 4 hospitals, using the same protocol.

No serious adverse events were recorded during or after IVDST.

### Assays

Note that the Research Resource Identifiers (RRIDs) for assays used in this study are hyperlinked to SciCrunch. The cortisol (ADVIA Centaur, Siemens Cat# 04610138, RRID:AB_3717364, https://scicrunch.org/resolver/RRID:AB_3717364) and ACTH (Immulite 2000, Siemens Cat# L2KAC2, RRID:AB_2783635, https://scicrunch.org/resolver/RRID:AB_2783635) assays used in control subjects were previously published [19]. For the patients with CS and LPC, the samples were analyzed by commercially available assays in the Departments of Biochemistry located at each of the hospitals where the IVDST was performed. The cortisol and ACTH assays used between 1995 and 2007 and correlations between these assays were previously published [19]. After 2007, SVHM laboratory continued to use ADVIA Centaur cortisol assay until 2009 when it was changed to Architect (Abbott Cat# 8D15, RRID:AB_2783639, https://scicrunch.org/resolver/RRID:AB_2783639); method comparison using regression analysis showed that Architect = (0.82×ADVIA Centaur) − 6.1, with correlation coefficient >0.9 (unpublished laboratory observation). At AH, Architect cortisol assay was used until 2019, and then Alinity (Abbot Cat# 08P33, RRID:AB_3717365, https://scicrunch.org/resolver/RRID:AB_3717365); method comparison using regression analysis showed that Alinity = (0.95×Architect) + 12.5 (unpublished laboratory observation). During 2007 to 2024, Immulite 2000 (Siemens Cat# LKCO2, RRID:AB_2810257, https://scicrunch.org/resolver/RRID:AB_2810257) cortisol assay was used at RHH, and Access cortisol (Beckman Coulter Cat# 33600, RRID:AB_2802133, https://scicrunch.org/resolver/RRID:AB_2802133) cortisol assay was used at PAH. ACTH Immulite 2000 assay was used at 3 centers (SVHM, RHH, PAH) during 2007-2024, while AH used Roche platform (Roche Diagnostics, Switzerland, Roche Cat# 03255751 190, RRID:AB_2783634, https://scicrunch.org/resolver/RRID:AB_2783634).

### Statistical Analysis

Results are presented as the mean ± standard error of the mean (SEM). For statistical purposes, the value corresponding to the limit of detection of assays was used for undetectable concentrations. Statistical analyses were performed using Prism version 10 (GraphPad). Sensitivity, specificity, and receiver-operating characteristic curves (ROC) were determined according to standard statistical methods. In the diagnosis of CS, the combined results from CD, EAS, and AC groups were considered to be a positive diagnosis, and combined results from the control and LPC groups were considered to be a negative diagnosis.

### Ethics Approval

The study was approved by the institutional human research ethics committees at the 4 participating hospitals.

## Results

**Figure 2.**
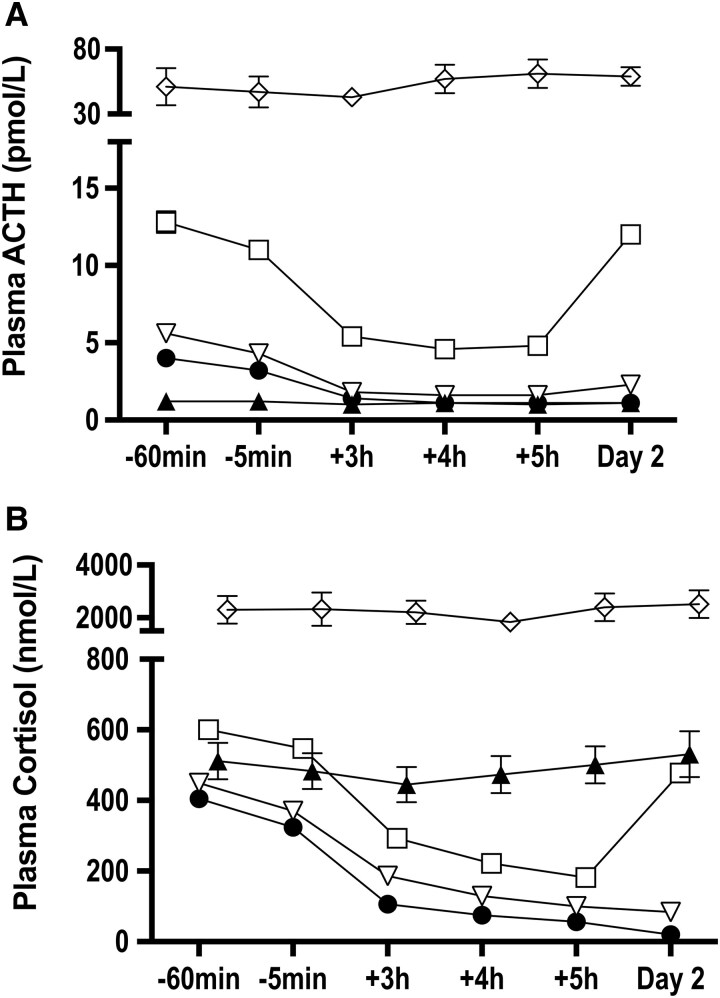
Mean (± SEM) ACTH (pmol/L) (A) and plasma cortisol (nmol/L) (B) during the intravenous dexamethasone suppression test in control group (⬤), LPC (▽), CD (□), EAS (◊), and AC (▴). Abbreviations: AC, adrenal Cushing; ACTH, adrenocorticotropic hormone; CD, Cushing disease; EAS, ectopic ACTH syndrome; LPC, low probability of Cushing syndrome.

Figure 2 shows the mean (± SEM) ACTH (a) and cortisol (b) levels. Table 2 shows the mean (±SEM) ACTH and cortisol levels at baseline, +5 hours and on Day 2. Control and LPC groups showed suppression of ACTH and cortisol at +5 hours which was maintained on Day 2. In the CD group, partial suppression of ACTH and cortisol at +5 hours was followed by rebound on Day 2. In patients with EAS, mean ACTH and cortisol levels were very high at baseline and not suppressed by dexamethasone. In the AC group, there was no suppression of mean cortisol following dexamethasone; the mean ACTH levels were 1.2 pmol/L or less throughout.

**Table 2.** Mean (±SEM) ACTH and cortisol levels at baseline, at +5 hours, and on day 2

	ACTH (pmol/L)			Cortisol (nmol/L)		
	Baseline	+5 hours	Day 2	Baseline	+5 hours	Day 2
Control	4.0 ± 0.4	1.1 ± 0.03	1.1 ± 0.05	405 ± 19	56 ± 4	20 ± 2
LPC	5.6 ± 0.6	1.6 ± 0.2	2.3 ± 0.3	449 ± 18	100 ± 8	84 ± 10
CD	13 ± 0.7	4.8 ± 0.5	12 ± 0.5	600 ± 20	182 ± 12	478 ± 22
EAS	51 ± 14	61 ± 11	59 ± 7	2308 ± 533	2403 ± 528	2517 ± 522
AC	1.2 ± 0.1	1.0 ± 0.1	1.1 ± 0.1	511 ± 52	501 ± 53	511 ± 65

Abbreviations: AC, adrenal Cushing syndrome; ACTH, adrenocorticotropin; CD, Cushing disease; EAS, ectopic adrenocorticotropin syndrome; LPC, low probability of Cushing syndrome; SEM, standard error of mean.

### The Diagnosis of Cushing Syndrome

**Figure 3.**
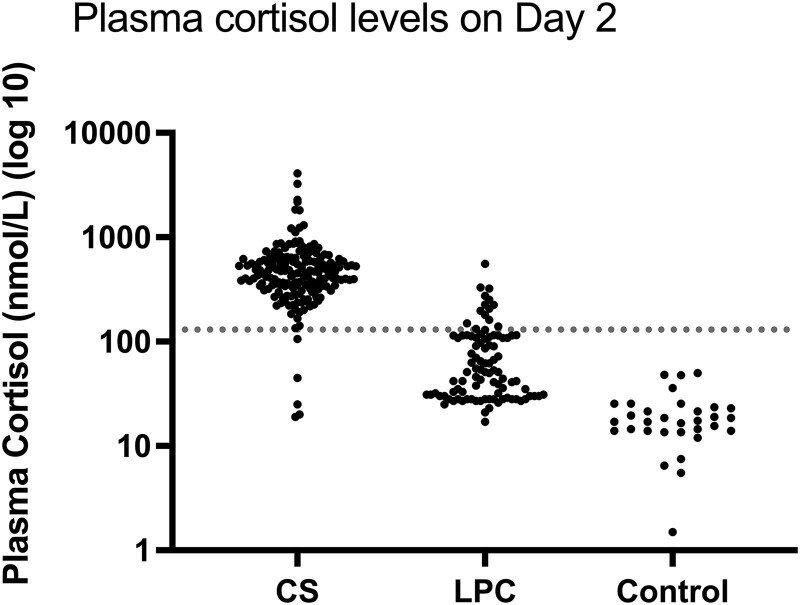
Day 2 plasma cortisol (nmol/L) in 173 patients with Cushing syndrome, 97 with low probability of Cushing syndrome and 32 control subjects. The dotted line represents the Day 2 cortisol cutoff level of 130 nmol/L. Abbreviations: CS, Cushing syndrome; LPC, low probability of Cushing syndrome.

Figure 3 shows the Day 2 cortisol levels in CS, compared to LPC and control group. In CS, Day 2 cortisol was >130 nmol/L in 166 of 171 patients (97%). During the first 14 years of IVDST (1995-2009), Day 2 cortisol was >130 nmol/L in 90 of 90 patients (100%) with CS. Between 2010 and 2024, Day 2 cortisol was >130 nmol/L in 76 of 81 (94%) patients with CS; false negative results were found in 5 patients with CD, of whom 4 had Day 2 cortisol levels <50 nmol/L (cortisol samples were measured by Architect in 2 patients and Access in 2 patients), and 1 patient had Day 2 cortisol between 100 and 130 nmol/L (measured by Architect). Of 140 patients, 3 (2%) in the CD group demonstrated cyclicity, defined as at least 2 peaks of hypercortisolism, interspersed with at least 1 trough of eucortisolism, based on screening test results for CS (24-hour UFC and/or LNSC); 2 of 3 patients with cyclic CD underwent IVDST during an eucortisolemic phase and had Day 2 cortisol levels <50 nmol/L, and 1 patient with cyclic CD underwent IVDST during a hypercortisolemic phase and had Day 2 cortisol level >130 nmol/L following IVDST. Of the 97 patients with LPC, 17 patients had Day 2 cortisol levels which were >130 nmol/L, including 5 patients with active depression; of the 17 false positive results in the LPC group, 4 patients (24%) had Day 2 cortisol levels between 131 and 160 nmol/L, and 13 patients (76%) had Day 2 cortisol levels >160 nmol/L (range, 161-557 nmol/L).

Analysis of the ROC curve demonstrated that the Day 2 cortisol cutoff of >130 nmol/L had a sensitivity of 97% and a specificity of 87% in the diagnosis of CS; at this cutoff, positive predictive value (PPV) was 91% and negative predictive value (NPV) was 96%. The lower cortisol cutoff of 100 nmol/L yielded sensitivity of 98%, specificity of 81%, PPV of 86%, and NPV of 95%.

We also evaluated the Day 2 ACTH diagnostic cutoff levels. The AC group was excluded from this analysis based on low or undetectable Day 2 ACTH levels in 88% (which were indistinguishable from control subjects), unavailable ACTH results in 4% and ACTH assay interference in 8%; these 2 patients had elevated ACTH levels on Immulite 2000 assay which was subsequently undetectable at <2.2 pmol/L on second assay platform, and 1 of these 2 patients also had other tests to confirm assay interference, including recovery after polyethylene glycol (PEG), serial dilution, and heterophil blocking reagent. Day 2 ACTH results were unavailable in 2 patients with CD (1%), 1 patient with EAS (20%), and 4 patients with LPC (4%). After excluding the AC group and patients with unavailable results, a Day 2 ACTH cutoff of 2.2 pmol/L yielded a sensitivity of 98%, a specificity of 75%, a PPV of 82%, and a NPV of 97% (data not shown). Using an ACTH cutoff of 3.3 pmol/L yielded a sensitivity of 94%, a specificity of 89%, a PPV of 91%, and a NPV of 93%.

### Differential Diagnosis of Cushing Syndrome

**Figure 4.**
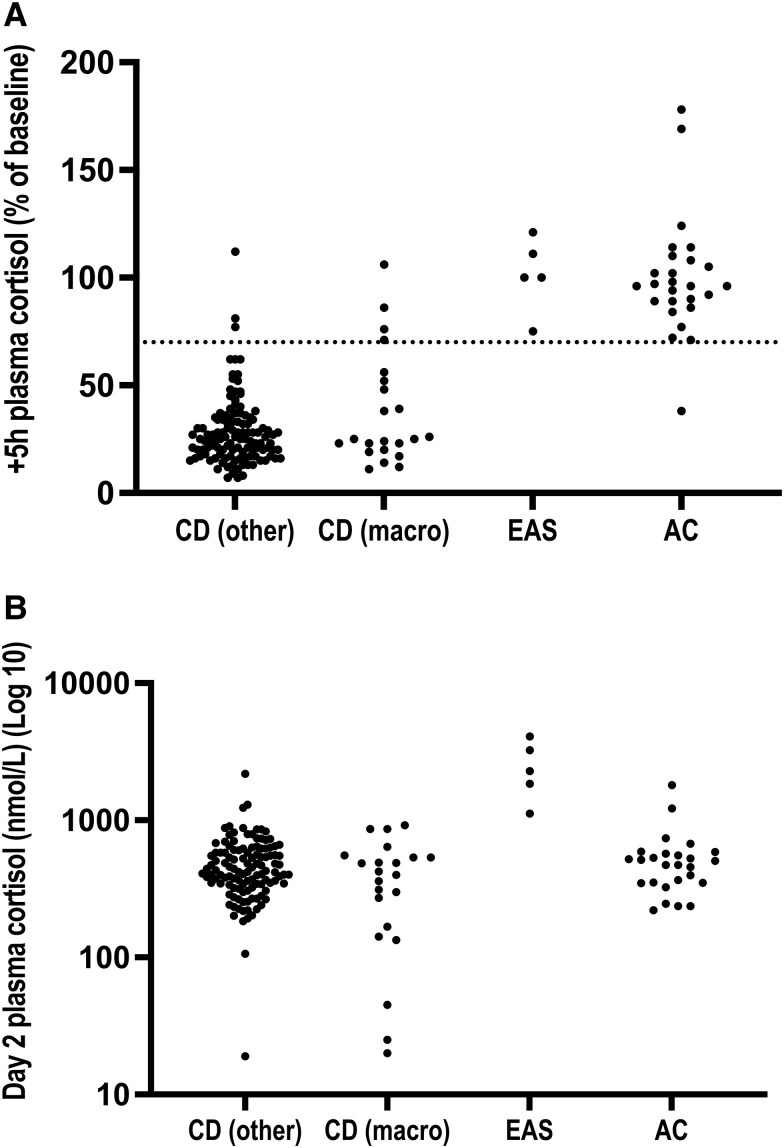
A, Percentage of baseline plasma cortisol levels at +5 hours according to the etiology of the Cushing syndrome. The dotted line represents the threshold of 70%. B, Day 2 plasma cortisol (nmol/L) levels according to the etiology of CS. The CD (other) group includes 100 patients (71% of CD data) with a pituitary microadenoma (adenoma <10 mm) and 18 patients (13% of CD data) in whom the MRI pituitary report was unavailable; CD (macro) consisted of 22 patients (16% of CD data) with a pituitary macroadenoma (adenoma >10 mm). Abbreviations: AC, adrenal Cushing; EAS, ectopic adrenocorticotropin syndrome.

We also evaluated the utility of IVDST in the differential diagnosis of CS. Figure 4A shows the individual plasma cortisol values at +5 hours calculated as a percentage of their baseline values, according to the etiology of CS. In CD, the mean +5 hours cortisol was 30% the baseline (range, 7%-112%). There were 3 types of cortisol patterns in the CD group overall, with the predominance of a partial suppression of +5 hours cortisol to <70% of the baseline, followed by rebound hypercortisolism on Day 2 in 125 of 140 patients (89%) (CD pattern *a*, the typical “pituitary” pattern). Of 140 patients, 7 (5%) with CD did not suppress the +5 hours cortisol to <70% of the baseline (CD pattern *b*). Of 140 patients, 8 (6%) with CD demonstrated suppression of +5 hours cortisol to <70% of the baseline without rebound hypercortisolism on Day 2 (CD pattern *c*); 2 of 8 patients in the CD pattern *c* group had the IVDST during the eucortisolemic phase of cyclic CD. Of the 100 patients with pituitary microadenoma in the CD group, 95 patients (95%) had cortisol responses which followed CD pattern *a*, 2 patients (2%) followed CD pattern *b*, and 3 patients (3%) followed CD pattern *c*. Of the 22 patients with pituitary macroadenoma in the CD group, 14 patients (64%) followed CD pattern *a*, 4 patients followed CD pattern *b* (18%), and 4 patients (18%) followed CD pattern *c*. In 5 patients with EAS, there was no suppression of +5 hours cortisol (mean cortisol, 101%, range 75%-121% of the baseline cortisol) (Fig. 4A). In the 26 patients with AC, the mean +5 hours cortisol was 100% of the baseline (range, 38%-178%); 1 patient demonstrated cortisol suppression to <70% at +5 hours (Fig 4A).

Day 2 cortisol levels did not discriminate CD from other causes of CS (Fig. 4B). In EAS, Day 2 cortisol levels were between 1120 and 4086 nmol/L; 2 patients with EAS and 3 patients with CD had Day 2 cortisol levels that overlapped between 1120 and 2200 nmol/L range.

## Discussion

This is the largest study examining the IVDST as a second-line diagnostic test to differentiate CS from those who do not have CS (control and LPC groups). This study demonstrated that a Day 2 cortisol cutoff of >130 nmol/L provided a sensitivity of 97% and a specificity of 87% in the diagnosis of CS. False negative Day 2 cortisol results occurred in 2 of 3 patients with cyclic CD when their IVDST was performed during the eucortisolemic phase; in 1 of 3 patients with cyclic CD, Day 2 cortisol was >130 nmol/L when the IVDST was performed during a hypercortisolemic phase. Patients with cyclic CS have phases of biochemical hypercortisolism (at least 2 peaks) interspersed with periods of eucortisolism or even hypocortisolism (at least 1 trough) [7, 8]. A recent systemic review reported that cyclic CS could account for 7% to 21% of patients with CD [7]. The consensus update of the Pituitary Society recommended that in cyclic CD, dynamic or localization testing should be preceded by LNSC or UFC [21]. Consistent with this recommendation, we now advise repeating LNSC or 24 hours UFC to confirm the active phase, within 24 to 48 hours before IVDST in patients with suspected cyclic CS. False positive results occurred in 5 of 5 patients with active depression using the Day 2 cortisol cutoff of 130 nmol/L. Previous studies reported abnormal responses to DST and elevated UFC in patients with depression [22] which is associated with increased HPA axis activity [9]. After the exclusion of 2 patients with cyclic CD who had the IVDST during an eucortisolemic phase and 5 patients with active depression, Day 2 cortisol cutoff of 130 nmol/L yielded sensitivity of 97% and specificity of 86%. Day 2 ACTH cutoff of 2.2 pmol/L yielded a high false positive rate of 25% and should not be used as a sole diagnostic criterion. Overall, the originally proposed Day 2 cortisol cutoff of >130 nmol/L [19] has high sensitivity without unduly compromising specificity. This cutoff has held up over time despite the use of different cortisol assays across the 4 centers.

At our centers, the IVDST was used primarily as a second-line diagnostic test in potential CS in patients with abnormal or discordant first-line screening test results, such as the 24-hour UFC, 1-mg DST, and LNSC, which were requested at the discretion of their referring doctors. We do not advocate the IVDST as a screening test. Although data on screening test results were incomplete, at least 12% of patients with noncyclic CS were documented to have discordant results; these patients had Day 2 cortisol levels following IVDST which were consistent with CS, prompting further investigation and treatment. At our centers, other second-line diagnostic tests, such as the midnight serum cortisol or the dexamethasone-suppressed corticotropin-releasing hormone stimulation (Dex-CRH) test [23], were performed infrequently. Thus, it was not possible to directly compare the diagnostic performance of IVDST with these established diagnostic tests in the same group of patients in our retrospective study. Collection of midnight plasma cortisol requires an inpatient admission which is usually impractical. Dex-CRH test requires oral dexamethasone to be given in doses of 0.5 mg, 6-hourly over 48 hours, which is followed by an intravenous administration of CRH (1 μg/kg). Overall, the Dex-CRH test yielded sensitivity of 97% and specificity of 92% in a recent meta-analysis of 9 studies which utilized various cortisol and/or ACTH cutoff values [24]. There has been limited availability of CRH worldwide, which was compounded by the cessation of CRH production by Ferring Pharmaceuticals from December 2022 [24]. Currently, CRH vials may be sourced from other companies in Japan or Switzerland, but whether CRH will be available in appropriate amounts for reasonable costs and over long periods is unknown [25]. Although we do not claim superiority of the IVDST based on the available data, our findings indicate it is a reliable outpatient-based second-line diagnostic test to distinguish CS from non-CS, particularly given the ongoing shortage of CRH. We propose that in clinical practice, the IVDST should be used in patients with discordant first-line screening tests or during a hypercortisolemic phase of suspected cyclic CS, and then proceeding to investigations to determine the cause of CS when the Day 2 cortisol is above 130 nmol/L during the IVDST.

Distinguishing between mild CS and those with clinical and/or biochemical features that mimic CS can be difficult [26]. Our study had a large number of patients in the LPC group, which included patients with obesity, diabetes mellitus, hypertension, dyslipidemia, depression, or hirsutism; the LPC group was analogous to physiological or non-neoplastic hypercortisolism [9], formerly known as pseudo-CS [26]. The LPC group was an important comparison group as they were initially investigated for suspected CS, but subsequent follow-up over a 2-year period revealed no progression in the clinical features of CS. Consistent with our previous study [19], follow-up information was used to distinguish LPC from CS, rather than the response to the IVDST, as the latter approach would have falsely increased the specificity of the test.

Differentiating between pituitary and ectopic sources of excess ACTH secretion can pose a considerable challenge. Our study included many patients with surgically proven CD, of whom 89% demonstrated the typical “pituitary” pattern of response to the IVDST, specifically, a partial suppression of +5 hours cortisol to <70% of the baseline, which was followed by rebound hypercortisolism on Day 2. Previous studies reported that the early escape of plasma cortisol from suppressive effect of 4 mg of intravenous dexamethasone was a consistent biochemical finding in CD [14, 18]. However, our study showed that Day 2 cortisol suppression was maintained (no rebound hypercortisolism) after the initial partial suppression at +5 hours in a small subgroup of CD (6%); 2 of 8 patients in this CD subgroup had the IVDST during the eucortisolemic phase of cyclic CD. There was an overlap between 5% of patients with CD and 100% of EAS who did not demonstrate partial suppression of +5 hours cortisol to <70% of the baseline. While there are no studies on the in vitro responses of corticotroph tumors to intravenous dexamethasone (1 mg/h for 4 hours), this overlap likely reflects physiological differences in a small number of patients with CD who do not demonstrate the typical partial responsiveness to the negative feedback effects of dexamethasone. It is possible that glucocorticoid-induced inhibition of the HPA axis is disrupted in some patients with CD, particularly as corticotroph tumors enlarge [8]. In our study, four of 22 patients (18%) with pituitary macroadenoma in the CD group did not suppress +5 hours cortisol to <70% of the baseline. It has been recognized that patients with macroadenomas show relative resistance to high-dose dexamethasone [27]. Given these considerations and the small number of EAS in our study, we do not advocate the use of IVDST alone in the differential diagnosis of ACTH-dependent CS, and oral high-dose DST (HDDST), inferior petrosal sinus sampling, and functional imaging remain necessary complementary tools. HDDST is most used in the world to differentiate CD from EAS [28]. Ceccato et al performed a meta-analysis of 43 studies which reported the use of HDDST, comprising over 3000 patients with ACTH-dependent CS, and the most commonly used schedules were 8-mg overnight HDDST or 2 mg of dexamethasone 6-hourly for 2 days (48-hour HDDST); the overall sensitivity and specificity of the serum cortisol suppression after HDDST were 81% and 84%, respectively, in differentiating CD from EAS [28]. The 48-hour HDDST has largely been replaced by the 8-mg overnight HDDST for convenience and to reduce issues with medication adherence [29]. Recent studies on the 8-mg HDDST demonstrated sensitivity and specificity rates between 79% and 80% and between 77% and 78%, respectively, in differentiating CD from EAS [29, 30], a diagnostic performance similar to the pretest probability of CD [31] which accounts for 80% to 90% of ACTH-dependent CS [32]. The oral HDDST (48-hour or 8 mg overnight schedules) were performed rarely at our centers, and therefore, it was not possible to directly compare the performance of IVDST with oral HDDST in the differential diagnosis of ACTH-dependent CS. However, when IVDST is primarily used as a diagnostic test, it has a secondary benefit in providing additional information on pattern of cortisol response; the typical “pituitary” pattern was found in 95% with patients surgically proven CD who had a pituitary microadenoma pre-operatively. Future directions include comparison of the IVDST with 8-mg overnight HDDST to distinguish between CD and EAS.

Low basal plasma ACTH of <10 pg/mL (<2.2 pmol/L) indicates ACTH-independent CS [1], and IVDST is not usually required in this situation. Indeterminate ACTH levels between 10 and 20 pg/mL (2.2-4.4 pmol/L) require additional testing [1]. In our study, 2 patients with AC with known adrenal adenoma were initially investigated for possible ACTH-dependent CS with IVDST, based on falsely elevated ACTH levels (performed on the Immulite platform) which were subsequently found to be due to assay interference, as shown by low ACTH levels of <2.2 pmol/L on a second assay platform; 1 of these 2 patients had other laboratory tests to confirm ACTH assay interference. Immulite ACTH assay was initially used in several case reports of spurious results [33-35], although interferences can occur rarely with any ACTH immunoassays [36]. While no single test is considered to be the “best” for identifying ACTH assay interference, a straightforward technique is to analyze the sample with an alternative assay as there is a high probability of interference if the data obtained on different platforms are markedly inconsistent [33, 35].

Our study excluded 6 patients with mild autonomous cortisol secretion (MACS) which is a separate condition to AC based on its different biology and natural history [37]. The risk of progression from MACS to overt CS is rare (<1%) [37]. We also excluded 1 patient with subclinical CD to focus our study on the diagnosis and differential diagnosis of clinically overt CS.

One of the limitations of our study was the use of different laboratories and assays in generating the retrospective CS and LPC data, which may compromise the precision that can be achieved using one methodology and measuring samples in the same batch [38]. In our study, it was not feasible to perform assay method comparison studies retrospectively using the same patient samples, given that the blood samples were discarded by the hospital laboratories after 1 week, in addition to the prohibitive cost of recommissioning old assay platforms. Roberts and Roberts [39] reported good concordance between 5 automated serum cortisol immunoassays, but subsequent studies have reported variability in the cortisol cutoff values after short Synacthen testing [40], insulin tolerance testing [41], and 1-mg DST [42] with the use of newer cortisol-specific assays. El-Farhan et al reported that compared to gas-chromatography–mass spectrometry, the bias ratios for modern cortisol immunoassays, including Centaur, Architect, Immulite 2000, and Access (which were used in our hospital laboratories during the IVDST study), were between 1.04 and 1.15 in male patients and between 1.00 and 1.11 in female patients not taking the oral contraceptive pill [40]. While we acknowledge that the absolute cortisol cutoff may change with different assay methods, our study demonstrated that the Day 2 cortisol cutoff of 130 nmol/L maintained an optimal diagnostic sensitivity, despite changes in cortisol assays over 30 years. Of 5 patients with CD with false negative results, 4 had Day 2 cortisol levels <50 nmol/L (using newer cortisol assays) which were far below the cutoff of 130 nmol/L and therefore, these results were unlikely to be due to changes in assay sensitivity over time, but partly due to other factors, such as the IVDST performed during an eucortisolemic phase of cyclic CD in 2 patients. Reducing the Day 2 cortisol cutoff to 100 nmol/L diagnosed only one more case of CD. Clinical judgment and index of suspicion for CS are important [21, 40], particularly in cases of IVDST results which are close to the diagnostic cutoff points. Although ACTH assays were not directly compared in our study, a recent publication reported a high correlation between Immulite and Roche ACTH assays based on linear regression analysis [43].

A limitation of our study was that dexamethasone levels were not measured during the IVDST as dexamethasone assays were unavailable at our centers across the 30-year study duration. Therefore, we cannot exclude interpersonal variability in the metabolism of intravenous dexamethasone. However, intravenous administration of dexamethasone avoids the issues with poor adherence or variable gastrointestinal absorption of oral dexamethasone which are major factors which can affect dexamethasone levels. Furthermore, our study excluded patients who were on anticonvulsant medications that induce hepatic clearance of dexamethasone and cause false positive results in any DST. Further studies are required to establish normative data on dexamethasone level reference ranges at +5 hours and Day 2 after 4 mg of intravenous dexamethasone which may be helpful in cases which do not follow the expected degree of cortisol suppression or pattern.

In conclusion, our study has demonstrated that IVDST is a highly sensitive second-line diagnostic test for CS. We suggest that false negative results may occur when IVDST is performed during the eucortisolemic phase of cyclic CS. The specificity of 87% was lower than our previous study [19], highlighting the importance of long-term follow-up of LPC and excluding patients with active depression. The small sample size for EAS is a major limitation in the use of the IVDST as the sole dynamic test in the differential diagnosis of ACTH-dependent CS. For the future, we propose a simplified IVDST protocol, measuring plasma cortisol before the start of the infusion at −60 minutes, and then at −5 minutes, +5 hours and 2 samples on Day 2 (+23 and +23.5 hours).

## Data Availability

Some or all datasets generated during and/or analyzed during the current study are not publicly available but are available from the corresponding author on reasonable request.

## Abbreviations

AC: adrenal Cushing syndrome
ACTH: adrenocorticotropin
AH: Alfred Hospital
CD: Cushing disease
CRH: corticotropin-releasing hormone
CS: Cushing syndrome
Dex-CRH: dexamethasone-suppressed corticotropin-releasing hormone stimulation
DST: dexamethasone suppression test
EAS: ectopic adrenocorticotropin syndrome
HDDST: high-dose dexamethasone suppression test
IVDST: intravenous dexamethasone suppression test
LNSC: late-night salivary cortisol
LPC: low probability of Cushing syndrome
NPV: negative predictive value
PAH: Princess Alexandra Hospital
PPV: positive predictive value
RHH: Royal Hobart Hospital
SVHM: St. Vincent’s Hospital, Melbourne
UFC: urinary free cortisol
